# Supplementary material for: Complexome profiling of the Chlamydomonas psb28 mutant reveals TEF5 as an early PSII assembly factor
Source: Plant Cell. 2025 Mar 17;37(6):koaf055. doi: 10.1093/plcell/koaf055 (PMC12164590; doi:10.1093/plcell/koaf055)
Supplement: koaf055_Supplementary_Data [file koaf055_supplementary_data.zip › psb28_interactive.zip!/plots/intensity/qualityPlots/peptides/_Cre16.g695100.t1.1.html]

### Cre16.g695100.t1.1

Trivial name:   
SeqCoverage [median]: 11.60  
Mass: 51.58 kDa  
  
 

| Sequence | MissedCleavage | Mass [Da] | wtIntensity | muIntensity | Score |
| --- | --- | --- | --- | --- | --- |
| AGSSSAAAGTGAGAK | 0 | 1162.56 | 693.70 | 475.16 | 52.69 |
| LPGVSNFNDTNK | 0 | 1304.64 | 465.46 | 643.93 | 81.02 |
| LYEEGR | 0 | 765.37 | 370.34 | 409.12 | 110.54 |
| MTHEQASLVK | 0 | 1142.58 | 393.53 | 0.00 | 73.50 |
| NNQDGQVNAFIVR | 0 | 1473.73 | 0.00 | 793.54 | 107.83 |
|  |  |  | 1923.0 | 2321.8 |  |
